# The Human Population: Accepting Species Limits

**DOI:** 10.1289/ehp.114-a17

**Published:** 2006-01

**Authors:** Steven Earl Salmony

**Affiliations:** Disability Determination Services, Raleigh, North Carolina, E-mail: sesalmony@aol.com

In “The Population Equation: Balancing What We Need with What We Have,” Dahl (2005) presented generally accepted thought and consensually validated data regarding the human population, even though he did not include an adequate scientific theory of absolute human population numbers. Dahl also appeared to confirm the wide agreement among scientists that it is difficult to make theoretical advances or conduct human population research because humankind is seen as essentially different from other species and the human world is viewed as being composed of many intricately connected things that interact in extremely complex ways. Therefore, the population dynamics of *Homo sapiens* are effectively relegated to the preternatural realm and are believed to include a number of factors that are so complicated and enormous as to be unsuitable for empirical research or else unknowable.

A theory of human population numbers that could objectively explain the increase and decrease of the human population would be useful. Perhaps correlation data from Hopfenberg and Pimentel (2001) and the recent mathematical formulation of this biologic phenomenon by Hopfenberg (2003) provide a basis for an apparently unexpected theoretical perspective. According to the empirical research (Hopfenberg 2003), human population growth is a rapidly cycling positive feedback loop in which food availability drives population growth and this growth in human numbers gives rise to the mistaken impression that food production needs to be increased even more.

The data of Hopfenberg (2003) and Hopfenberg and Pimentel (2001) indicate that the world’s human population—all segments of it—grows by approximately 2% per year, including more people with brown eyes and more with blue eyes; more tall people and more short people; and more people who grow up well fed and more who grow up hungry. We may or may not be reducing hunger by increasing food production; however, we are most certainly producing more and more hungry people. The evidence suggests that the remarkably successful efforts of humankind to increase food production to feed a growing population results in even greater increase in population numbers.

Hopfenberg and Pimentel (2001) pointed out that the perceived need to increase food production to feed a growing population is a misperception, a denial of the physical reality of the space–time dimension. If people are starving at a given moment in time, increasing food production cannot help them. Are these starving people supposed to be waiting for sowing, growing, and reaping to be completed? Are they supposed to wait for surpluses to reach them? Without food they would die. In such circumstances, increasing food production for people who are starving is like tossing parachutes to people who have already fallen out of the airplane—the produced food arrives too late. However, this does not mean human starvation is inevitable.

If this view of the human population is somehow correct, then human population dynamics are not biologically different in essence from the population dynamics of other species (Hopfenberg and Pimentel 2001). We do not find hoards of starving roaches, birds, squirrels, alligators, or chimpanzees in the absence of food as we do in many civilized human communities today, because these nonhuman species are not annually increasing their own production of food. Among tribal peoples in remote original habitats, we do not find people starving. Like nonhuman species, “primitive” human beings live within the carrying capacity of their environment. History is replete with examples of early humans and other ancestors not increasing their food production annually, but rather living successfully off the land for thousands of years as hunters and gatherers of food. Before the agricultural revolution and the production of more food than was needed for immediate survival, human numbers supposedly could not grow beyond their environment’s physical capacity to sustain them because human population growth or decline is primarily a function of food availability (Hopfenberg 2003; Hopfenberg and Pimentel 2001).

Given its current scale and rate of growth, the human population worldwide has identifiable, potentially destructive ecological consequences. From this theoretical perspective, recent global human population growth can be understood as a primary causative factor of a range of phenomena including biodiversity loss and environmental degradation.
